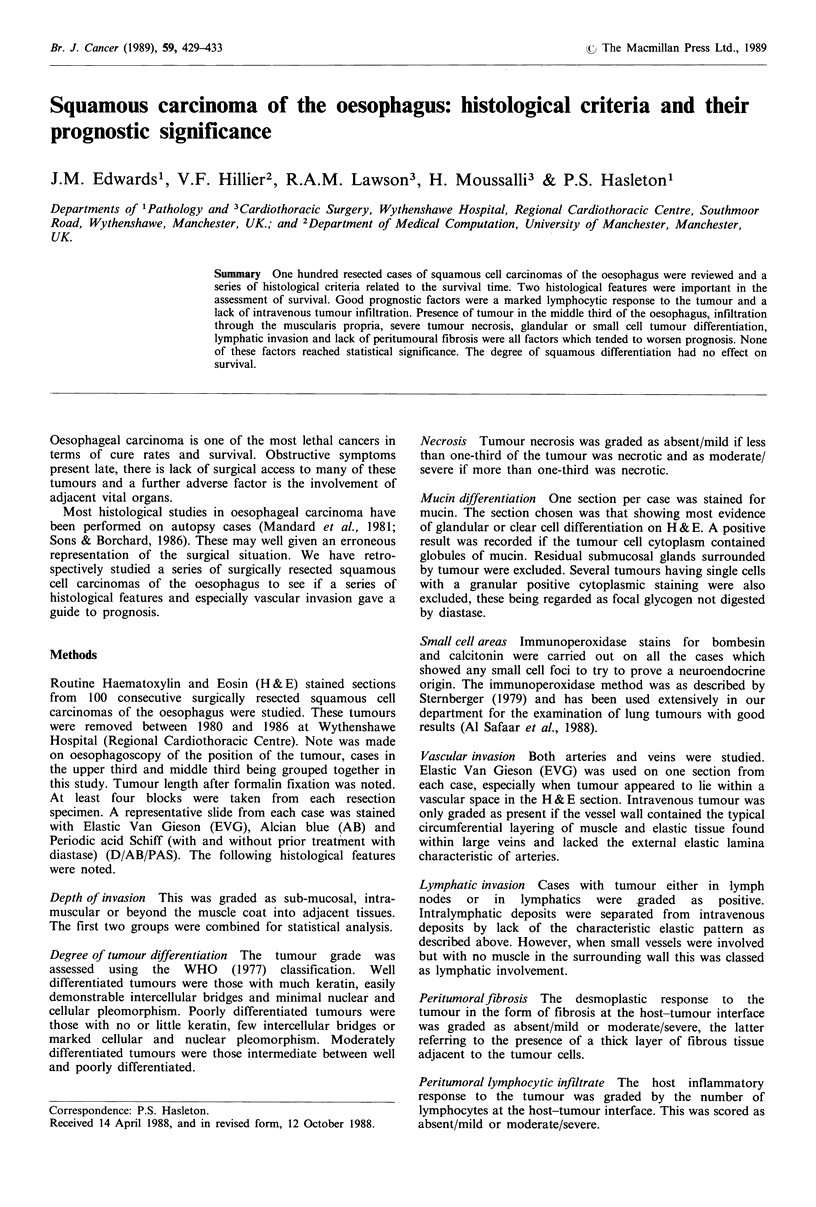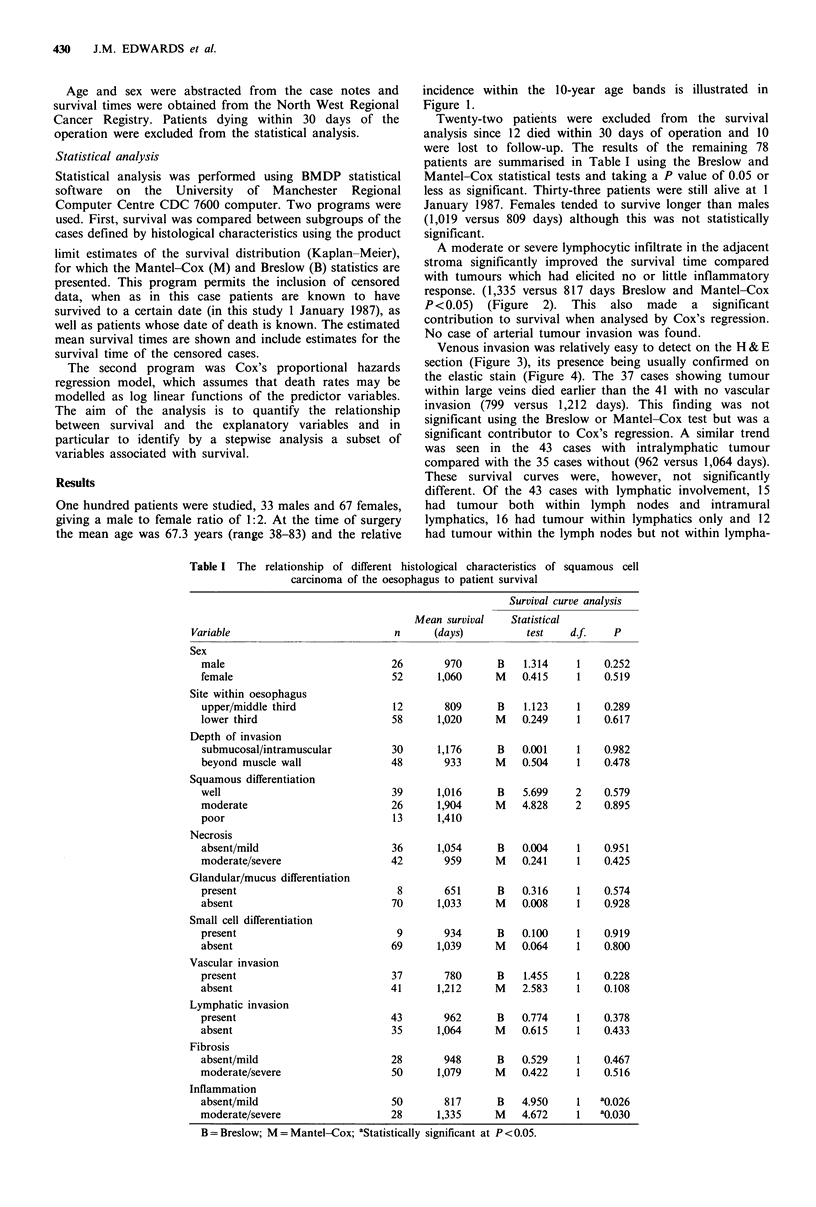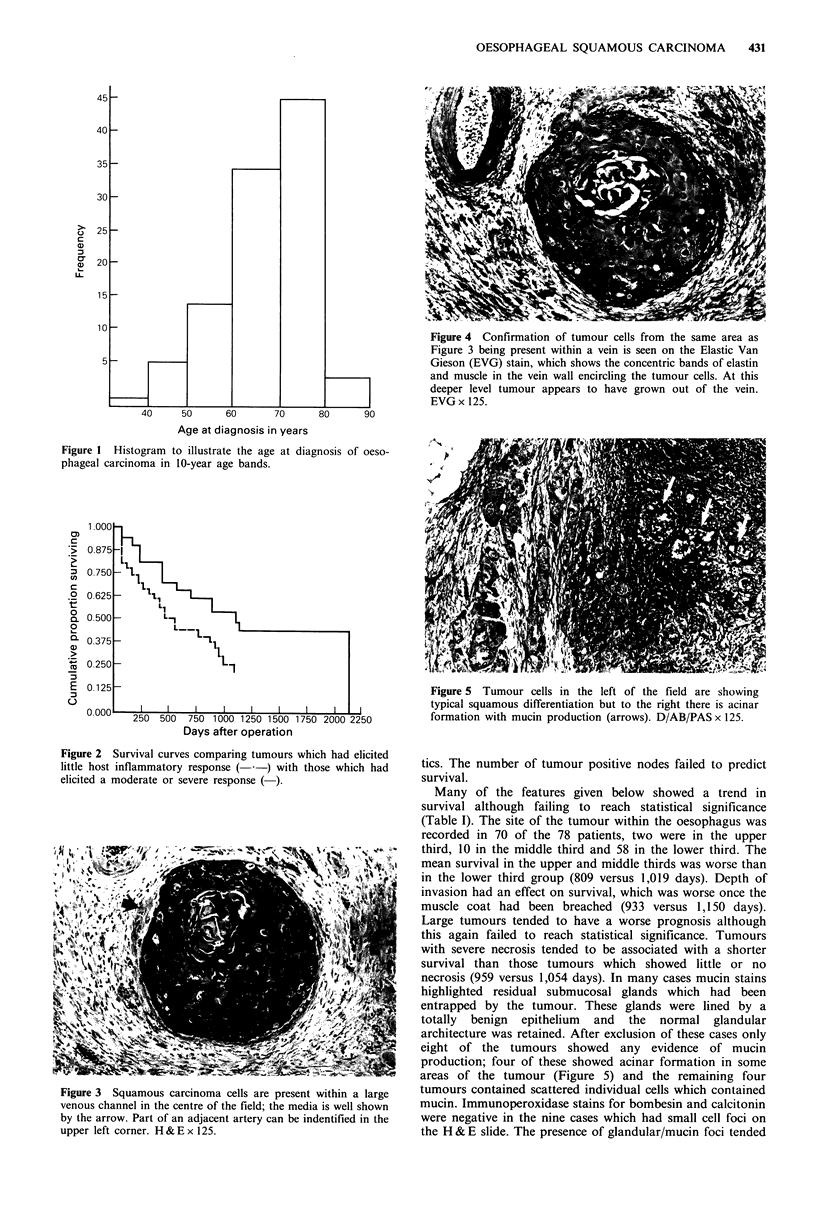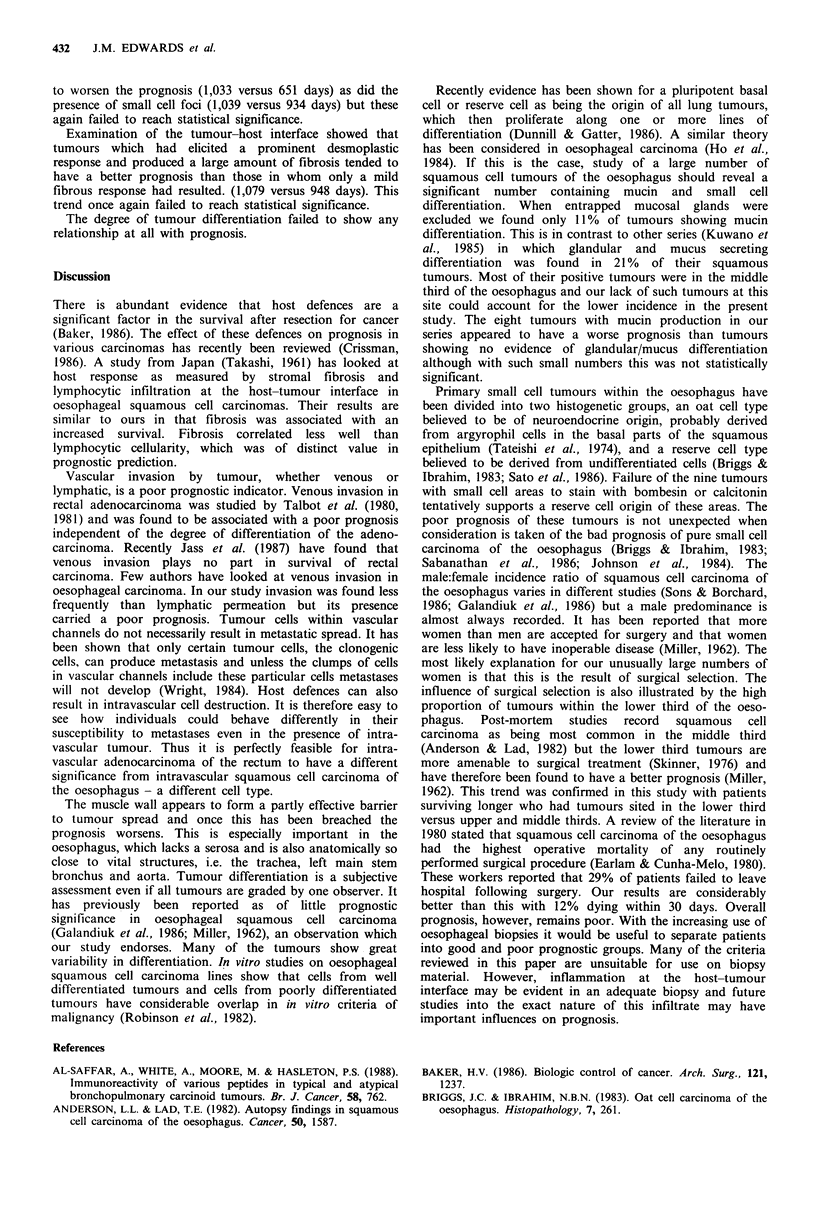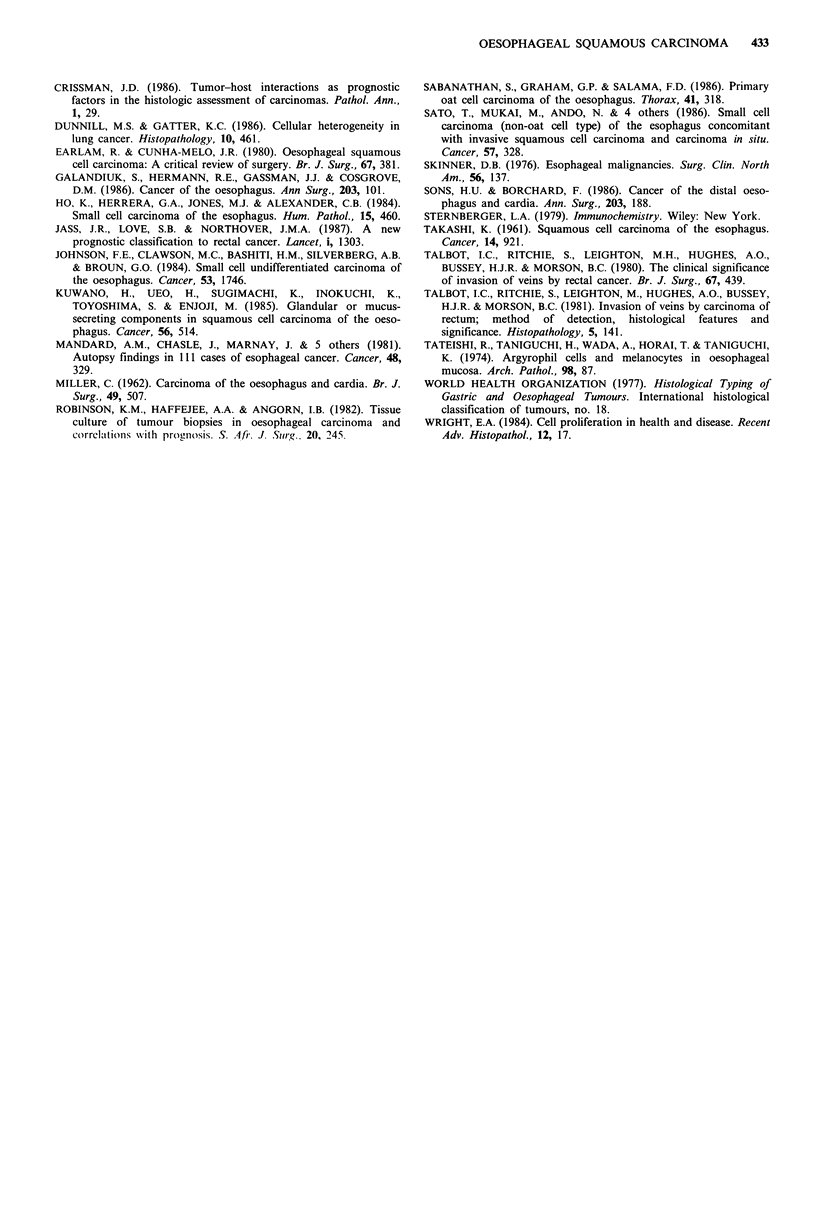# Squamous carcinoma of the oesophagus: histological criteria and their prognostic significance.

**DOI:** 10.1038/bjc.1989.87

**Published:** 1989-03

**Authors:** J. M. Edwards, V. F. Hillier, R. A. Lawson, H. Moussalli, P. S. Hasleton

**Affiliations:** Department of Pathology, Wythenshawe Hospital, Manchester, UK.

## Abstract

**Images:**


*Br. J. Cancer* (1989), **59**, 429–433 © The Macmillan Press Ltd., 1989

### Squamous carcinoma of the oesophagus: histological criteria and their prognostic significance

J.M. Edwards[1], V.F. Hillier[2], R.A.M. Lawson[3], H. Moussalli[3] & P.S. Hasleton[1]

*Departments of [1]Pathology and [3]Cardiothoracic Surgery, Wythenshawe Hospital, Regional Cardiothoracic Centre, Southmoor Road, Wythenshawe, Manchester, UK.; and [2]Department of Medical Computation, University of Manchester, Manchester, UK.*

**Summary** One hundred resected cases of squamous cell carcinomas of the oesophagus were reviewed and a series of histological criteria related to the survival time. Two histological features were important in the assessment of survival. Good prognostic factors were a marked lymphocytic response to the tumour and a lack of intravenous tumour infiltration. Presence of tumour in the middle third of the oesophagus, infiltration through the muscularis propria, severe tumour necrosis, glandular or small cell tumour differentiation, lymphatic invasion and lack of peritumoural fibrosis were all factors which tended to worsen prognosis. None of these factors reached statistical significance. The degree of squamous differentiation had no effect on survival.

Oesophageal carcinoma is one of the most lethal cancers in terms of cure rates and survival. Obstructive symptoms present late, there is lack of surgical access to many of these tumours and a further adverse factor is the involvement of adjacent vital organs.

Most histological studies in oesophageal carcinoma have been performed on autopsy cases (Mandard *et al.*, 1981; Sons & Borchard, 1986). These may well given an erroneous representation of the surgical situation. We have retrospectively studied a series of surgically resected squamous cell carcinomas of the oesophagus to see if a series of histological features and especially vascular invasion gave a guide to prognosis.

### Methods

Routine Haematoxylin and Eosin (H&E) stained sections from 100 consecutive surgically resected squamous cell carcinomas of the oesophagus were studied. These tumours were removed between 1980 and 1986 at Wythenshawe Hospital (Regional Cardiothoracic Centre). Note was made on oesophagoscopy of the position of the tumour, cases in the upper third and middle third being grouped together in this study. Tumour length after formalin fixation was noted. At least four blocks were taken from each resection specimen. A representative slide from each case was stained with Elastic Van Gieson (EVG), Alcian blue (AB) and Periodic acid Schiff (with and without prior treatment with diastase) (D/AB/PAS). The following histological features were noted.

*Depth of invasion* This was graded as sub-mucosal, intramuscular or beyond the muscle coat into adjacent tissues. The first two groups were combined for statistical analysis.

*Degree of tumour differentiation* The tumour grade was assessed using the WHO (1977) classification. Well differentiated tumours were those with much keratin, easily demonstrable intercellular bridges and minimal nuclear and cellular pleomorphism. Poorly differentiated tumours were those with no or little keratin, few intercellular bridges or marked cellular and nuclear pleomorphism. Moderately differentiated tumours were those intermediate between well and poorly differentiated.

Correspondence: P.S. Hasleton.
Received 14 April 1988, and in revised form, 12 October 1988.

*Necrosis* Tumour necrosis was graded as absent/mild if less than one-third of the tumour was necrotic and as moderate/severe if more than one-third was necrotic.

*Mucin differentiation* One section per case was stained for mucin. The section chosen was that showing most evidence of glandular or clear cell differentiation on H&E. A positive result was recorded if the tumour cell cytoplasm contained globules of mucin. Residual submucosal glands surrounded by tumour were excluded. Several tumours having single cells with a granular positive cytoplasmic staining were also excluded, these being regarded as focal glycogen not digested by diastase.

*Small cell areas* Immunoperoxidase stains for bombesin and calcitonin were carried out on all the cases which showed any small cell foci to try to prove a neuroendocrine origin. The immunoperoxidase method was as described by Sternberger (1979) and has been used extensively in our department for the examination of lung tumours with good results (Al Safaar *et al.*, 1988).

*Vascular invasion* Both arteries and veins were studied. Elastic Van Gieson (EVG) was used on one section from each case, especially when tumour appeared to lie within a vascular space in the H&E section. Intravenous tumour was only graded as present if the vessel wall contained the typical circumferential layering of muscle and elastic tissue found within large veins and lacked the external elastic lamina characteristic of arteries.

*Lymphatic invasion* Cases with tumour either in lymph nodes or in lymphatics were graded as positive. Intralymphatic deposits were separated from intravenous deposits by lack of the characteristic elastic pattern as described above. However, when small vessels were involved but with no muscle in the surrounding wall this was classed as lymphatic involvement.

*Peritumoral fibrosis* The desmoplastic response to the tumour in the form of fibrosis at the host–tumour interface was graded as absent/mild or moderate/severe, the latter referring to the presence of a thick layer of fibrous tissue adjacent to the tumour cells.

*Peritumoral lymphocytic infiltrate* The host inflammatory response to the tumour was graded by the number of lymphocytes at the host–tumour interface. This was scored as absent/mild or moderate/severe.

Age and sex were abstracted from the case notes and survival times were obtained from the North West Regional Cancer Registry. Patients dying within 30 days of the operation were excluded from the statistical analysis.

*Statistical analysis*

Statistical analysis was performed using BMDP statistical software on the University of Manchester Regional Computer Centre CDC 7600 computer. Two programs were used. First, survival was compared between subgroups of the cases defined by histological characteristics using the product limit estimates of the survival distribution (Kaplan–Meier), for which the Mantel–Cox (M) and Breslow (B) statistics are presented. This program permits the inclusion of censored data, when as in this case patients are known to have survived to a certain date (in this study 1 January 1987), as well as patients whose date of death is known. The estimated mean survival times are shown and include estimates for the survival time of the censored cases.

The second program was Cox's proportional hazards regression model, which assumes that death rates may be modelled as log linear functions of the predictor variables. The aim of the analysis is to quantify the relationship between survival and the explanatory variables and in particular to identify by a stepwise analysis a subset of variables associated with survival.

### Results

One hundred patients were studied, 33 males and 67 females, giving a male to female ratio of 1:2. At the time of surgery the mean age was 67.3 years (range 38–83) and the relative incidence within the 10-year age bands is illustrated in Figure 1.

Twenty-two patients were excluded from the survival analysis since 12 died within 30 days of operation and 10 were lost to follow-up. The results of the remaining 78 patients are summarised in Table I using the Breslow and Mantel–Cox statistical tests and taking a $P$ value of 0.05 or less as significant. Thirty-three patients were still alive at 1 January 1987. Females tended to survive longer than males (1,019 versus 809 days) although this was not statistically significant.

A moderate or severe lymphocytic infiltrate in the adjacent stroma significantly improved the survival time compared with tumours which had elicited no or little inflammatory response. (1,335 versus 817 days Breslow and Mantel–Cox $P<0.05$) (Figure 2). This also made a significant contribution to survival when analysed by Cox's regression. No case of arterial tumour invasion was found.

Venous invasion was relatively easy to detect on the H & E section (Figure 3), its presence being usually confirmed on the elastic stain (Figure 4). The 37 cases showing tumour within large veins died earlier than the 41 with no vascular invasion (799 versus 1,212 days). This finding was not significant using the Breslow or Mantel–Cox test but was a significant contributor to Cox's regression. A similar trend was seen in the 43 cases with intralymphatic tumour compared with the 35 cases without (962 versus 1,064 days). These survival curves were, however, not significantly different. Of the 43 cases with lymphatic involvement, 15 had tumour both within lymph nodes and intramural lymphatics, 16 had tumour within lymphatics only and 12 had tumour within the lymph nodes but not within lympha-

**Table I** The relationship of different histological characteristics of squamous cell carcinoma of the oesophagus to patient survival

| | | | *Survival curve analysis* | | | |
|---|---|---|---|---|---|---|
| *Variable* | *n* | *Mean survival (days)* | | *Statistical test* | *d.f.* | *P* |
| Sex | | | | | | |
| male | 26 | 970 | B | 1.314 | 1 | 0.252 |
| female | 52 | 1,060 | M | 0.415 | 1 | 0.519 |
| Site within oesophagus | | | | | | |
| upper/middle third | 12 | 809 | B | 1.123 | 1 | 0.289 |
| lower third | 58 | 1,020 | M | 0.249 | 1 | 0.617 |
| Depth of invasion | | | | | | |
| submucosal/intramuscular | 30 | 1,176 | B | 0.001 | 1 | 0.982 |
| beyond muscle wall | 48 | 933 | M | 0.504 | 1 | 0.478 |
| Squamous differentiation | | | | | | |
| well | 39 | 1,016 | B | 5.699 | 2 | 0.579 |
| moderate | 26 | 1,904 | M | 4.828 | 2 | 0.895 |
| poor | 13 | 1,410 | | | | |
| Necrosis | | | | | | |
| absent/mild | 36 | 1,054 | B | 0.004 | 1 | 0.951 |
| moderate/severe | 42 | 959 | M | 0.241 | 1 | 0.425 |
| Glandular/mucus differentiation | | | | | | |
| present | 8 | 651 | B | 0.316 | 1 | 0.574 |
| absent | 70 | 1,033 | M | 0.008 | 1 | 0.928 |
| Small cell differentiation | | | | | | |
| present | 9 | 934 | B | 0.100 | 1 | 0.919 |
| absent | 69 | 1,039 | M | 0.064 | 1 | 0.800 |
| Vascular invasion | | | | | | |
| present | 37 | 780 | B | 1.455 | 1 | 0.228 |
| absent | 41 | 1,212 | M | 2.583 | 1 | 0.108 |
| Lymphatic invasion | | | | | | |
| present | 43 | 962 | B | 0.774 | 1 | 0.378 |
| absent | 35 | 1,064 | M | 0.615 | 1 | 0.433 |
| Fibrosis | | | | | | |
| absent/mild | 28 | 948 | B | 0.529 | 1 | 0.467 |
| moderate/severe | 50 | 1,079 | M | 0.422 | 1 | 0.516 |
| Inflammation | | | | | | |
| absent/mild | 50 | 817 | B | 4.950 | 1 | [a]0.026 |
| moderate/severe | 28 | 1,335 | M | 4.672 | 1 | [a]0.030 |

B = Breslow; M = Mantel–Cox; [a]Statistically significant at $P<0.05$.

**Figure 1** Histogram to illustrate the age at diagnosis of oesophageal carcinoma in 10-year age bands.

**Figure 2** Survival curves comparing tumours which had elicited little host inflammatory response (—·—) with those which had elicited a moderate or severe response (—).

**Figure 3** Squamous carcinoma cells are present within a large venous channel in the centre of the field; the media is well shown by the arrow. Part of an adjacent artery can be indentified in the upper left corner. H & E × 125.

**Figure 4** Confirmation of tumour cells from the same area as Figure 3 being present within a vein is seen on the Elastic Van Gieson (EVG) stain, which shows the concentric bands of elastin and muscle in the vein wall encircling the tumour cells. At this deeper level tumour appears to have grown out of the vein. EVG × 125.

**Figure 5** Tumour cells in the left of the field are showing typical squamous differentiation but to the right there is acinar formation with mucin production (arrows). D/AB/PAS × 125.

tics. The number of tumour positive nodes failed to predict survival.

Many of the features given below showed a trend in survival although failing to reach statistical significance (Table I). The site of the tumour within the oesophagus was recorded in 70 of the 78 patients, two were in the upper third, 10 in the middle third and 58 in the lower third. The mean survival in the upper and middle thirds was worse than in the lower third group (809 versus 1,019 days). Depth of invasion had an effect on survival, which was worse once the muscle coat had been breached (933 versus 1,150 days). Large tumours tended to have a worse prognosis although this again failed to reach statistical significance. Tumours with severe necrosis tended to be associated with a shorter survival than those tumours which showed little or no necrosis (959 versus 1,054 days). In many cases mucin stains highlighted residual submucosal glands which had been entrapped by the tumour. These glands were lined by a totally benign epithelium and the normal glandular architecture was retained. After exclusion of these cases only eight of the tumours showed any evidence of mucin production; four of these showed acinar formation in some areas of the tumour (Figure 5) and the remaining four tumours contained scattered individual cells which contained mucin. Immunoperoxidase stains for bombesin and calcitonin were negative in the nine cases which had small cell foci on the H & E slide. The presence of glandular/mucin foci tended to worsen the prognosis (1,033 versus 651 days) as did the presence of small cell foci (1,039 versus 934 days) but these again failed to reach statistical significance.

Examination of the tumour–host interface showed that tumours which had elicited a prominent desmoplastic response and produced a large amount of fibrosis tended to have a better prognosis than those in whom only a mild fibrous response had resulted. (1,079 versus 948 days). This trend once again failed to reach statistical significance.

The degree of tumour differentiation failed to show any relationship at all with prognosis.

### Discussion

There is abundant evidence that host defences are a significant factor in the survival after resection for cancer (Baker, 1986). The effect of these defences on prognosis in various carcinomas has recently been reviewed (Crissman, 1986). A study from Japan (Takashi, 1961) has looked at host response as measured by stromal fibrosis and lymphocytic infiltration at the host–tumour interface in oesophageal squamous cell carcinomas. Their results are similar to ours in that fibrosis was associated with an increased survival. Fibrosis correlated less well than lymphocytic cellularity, which was of distinct value in prognostic prediction.

Vascular invasion by tumour, whether venous or lymphatic, is a poor prognostic indicator. Venous invasion in rectal adenocarcinoma was studied by Talbot *et al.* (1980, 1981) and was found to be associated with a poor prognosis independent of the degree of differentiation of the adenocarcinoma. Recently Jass *et al.* (1987) have found that venous invasion plays no part in survival of rectal carcinoma. Few authors have looked at venous invasion in oesophageal carcinoma. In our study invasion was found less frequently than lymphatic permeation but its presence carried a poor prognosis. Tumour cells within vascular channels do not necessarily result in metastatic spread. It has been shown that only certain tumour cells, the clonogenic cells, can produce metastasis and unless the clumps of cells in vascular channels include these particular cells metastases will not develop (Wright, 1984). Host defences can also result in intravascular cell destruction. It is therefore easy to see how individuals could behave differently in their susceptibility to metastases even in the presence of intravascular tumour. Thus it is perfectly feasible for intravascular adenocarcinoma of the rectum to have a different significance from intravascular squamous cell carcinoma of the oesophagus – a different cell type.

The muscle wall appears to form a partly effective barrier to tumour spread and once this has been breached the prognosis worsens. This is especially important in the oesophagus, which lacks a serosa and is also anatomically so close to vital structures, i.e. the trachea, left main stem bronchus and aorta. Tumour differentiation is a subjective assessment even if all tumours are graded by one observer. It has previously been reported as of little prognostic significance in oesophageal squamous cell carcinoma (Galandiuk *et al.*, 1986; Miller, 1962), an observation which our study endorses. Many of the tumours show great variability in differentiation. *In vitro* studies on oesophageal squamous cell carcinoma lines show that cells from well differentiated tumours and cells from poorly differentiated tumours have considerable overlap in *in vitro* criteria of malignancy (Robinson *et al.*, 1982).

Recently evidence has been shown for a pluripotent basal cell or reserve cell as being the origin of all lung tumours, which then proliferate along one or more lines of differentiation (Dunnill & Gatter, 1986). A similar theory has been considered in oesophageal carcinoma (Ho *et al.*, 1984). If this is the case, study of a large number of squamous cell tumours of the oesophagus should reveal a significant number containing mucin and small cell differentiation. When entrapped mucosal glands were excluded we found only 11% of tumours showing mucin differentiation. This is in contrast to other series (Kuwano *et al.*, 1985) in which glandular and mucus secreting differentiation was found in 21% of their squamous tumours. Most of their positive tumours were in the middle third of the oesophagus and our lack of such tumours at this site could account for the lower incidence in the present study. The eight tumours with mucin production in our series appeared to have a worse prognosis than tumours showing no evidence of glandular/mucus differentiation although with such small numbers this was not statistically significant.

Primary small cell tumours within the oesophagus have been divided into two histogenetic groups, an oat cell type believed to be of neuroendocrine origin, probably derived from argyrophil cells in the basal parts of the squamous epithelium (Tateishi *et al.*, 1974), and a reserve cell type believed to be derived from undifferentiated cells (Briggs & Ibrahim, 1983; Sato *et al.*, 1986). Failure of the nine tumours with small cell areas to stain with bombesin or calcitonin tentatively supports a reserve cell origin of these areas. The poor prognosis of these tumours is not unexpected when consideration is taken of the bad prognosis of pure small cell carcinoma of the oesophagus (Briggs & Ibrahim, 1983; Sabanathan *et al.*, 1986; Johnson *et al.*, 1984). The male:female incidence ratio of squamous cell carcinoma of the oesophagus varies in different studies (Sons & Borchard, 1986; Galandiuk *et al.*, 1986) but a male predominance is almost always recorded. It has been reported that more women than men are accepted for surgery and that women are less likely to have inoperable disease (Miller, 1962). The most likely explanation for our unusually large numbers of women is that this is the result of surgical selection. The influence of surgical selection is also illustrated by the high proportion of tumours within the lower third of the oesophagus. Post-mortem studies record squamous cell carcinoma as being most common in the middle third (Anderson & Lad, 1982) but the lower third tumours are more amenable to surgical treatment (Skinner, 1976) and have therefore been found to have a better prognosis (Miller, 1962). This trend was confirmed in this study with patients surviving longer who had tumours sited in the lower third versus upper and middle thirds. A review of the literature in 1980 stated that squamous cell carcinoma of the oesophagus had the highest operative mortality of any routinely performed surgical procedure (Earlam & Cunha-Melo, 1980). These workers reported that 29% of patients failed to leave hospital following surgery. Our results are considerably better than this with 12% dying within 30 days. Overall prognosis, however, remains poor. With the increasing use of oesophageal biopsies it would be useful to separate patients into good and poor prognostic groups. Many of the criteria reviewed in this paper are unsuitable for use on biopsy material. However, inflammation at the host–tumour interface may be evident in an adequate biopsy and future studies into the exact nature of this infiltrate may have important influences on prognosis.

### References

AL-SAFFAR, A., WHITE, A., MOORE, M. & HASLETON, P.S. (1988). Immunoreactivity of various peptides in typical and atypical bronchopulmonary carcinoid tumours. *Br. J. Cancer*, **58**, 762.

ANDERSON, L.L. & LAD, T.E. (1982). Autopsy findings in squamous cell carcinoma of the oesophagus. *Cancer*, **50**, 1587.

BAKER, H.V. (1986). Biologic control of cancer. *Arch. Surg.*, **121**, 1237.

BRIGGS, J.C. & IBRAHIM, N.B.N. (1983). Oat cell carcinoma of the oesophagus. *Histopathology*, **7**, 261.

CRISSMAN, J.D. (1986). Tumor–host interactions as prognostic factors in the histologic assessment of carcinomas. *Pathol. Ann.*, **1**, 29.

DUNNILL, M.S. & GATTER, K.C. (1986). Cellular heterogeneity in lung cancer. *Histopathology*, **10**, 461.

EARLAM, R. & CUNHA-MELO, J.R. (1980). Oesophageal squamous cell carcinoma: A critical review of surgery. *Br. J. Surg.*, **67**, 381.

GALANDIUK, S., HERMANN, R.E., GASSMAN, J.J. & COSGROVE, D.M. (1986). Cancer of the oesophagus. *Ann Surg.*, **203**, 101.

HO, K., HERRERA, G.A., JONES, M.J. & ALEXANDER, C.B. (1984). Small cell carcinoma of the esophagus. *Hum. Pathol.*, **15**, 460.

JASS, J.R., LOVE, S.B. & NORTHOVER, J.M.A. (1987). A new prognostic classification to rectal cancer. *Lancet*, **i**, 1303.

JOHNSON, F.E., CLAWSON, M.C., BASHITI, H.M., SILVERBERG, A.B. & BROUN, G.O. (1984). Small cell undifferentiated carcinoma of the oesophagus. *Cancer*, **53**, 1746.

KUWANO, H., UEO, H., SUGIMACHI, K., INOKUCHI, K., TOYOSHIMA, S. & ENJOJI, M. (1985). Glandular or mucus-secreting components in squamous cell carcinoma of the oesophagus. *Cancer*, **56**, 514.

MANDARD, A.M., CHASLE, J., MARNAY, J. & 5 others (1981). Autopsy findings in 111 cases of esophageal cancer. *Cancer*, **48**, 329.

MILLER, C. (1962). Carcinoma of the oesophagus and cardia. *Br. J. Surg.*, **49**, 507.

ROBINSON, K.M., HAFFEJEE, A.A. & ANGORN, I.B. (1982). Tissue culture of tumour biopsies in oesophageal carcinoma and correlations with prognosis. *S. Afr. J. Surg.*, **20**, 245.

SABANATHAN, S., GRAHAM, G.P. & SALAMA, F.D. (1986). Primary oat cell carcinoma of the oesophagus. *Thorax*, **41**, 318.

SATO, T., MUKAI, M., ANDO, N. & 4 others (1986). Small cell carcinoma (non-oat cell type) of the esophagus concomitant with invasive squamous cell carcinoma and carcinoma *in situ*. *Cancer*, **57**, 328.

SKINNER, D.B. (1976). Esophageal malignancies. *Surg. Clin. North Am.*, **56**, 137.

SONS, H.U. & BORCHARD, F. (1986). Cancer of the distal oesophagus and cardia. *Ann. Surg.*, **203**, 188.

STERNBERGER, L.A. (1979). *Immunochemistry*. Wiley: New York.

TAKASHI, K. (1961). Squamous cell carcinoma of the esophagus. *Cancer*, **14**, 921.

TALBOT, I.C., RITCHIE, S., LEIGHTON, M.H., HUGHES, A.O., BUSSEY, H.J.R. & MORSON, B.C. (1980). The clinical significance of invasion of veins by rectal cancer. *Br. J. Surg.*, **67**, 439.

TALBOT, I.C., RITCHIE, S., LEIGHTON, M., HUGHES, A.O., BUSSEY, H.J.R. & MORSON, B.C. (1981). Invasion of veins by carcinoma of rectum; method of detection, histological features and significance. *Histopathology*, **5**, 141.

TATEISHI, R., TANIGUCHI, H., WADA, A., HORAI, T. & TANIGUCHI, K. (1974). Argyrophil cells and melanocytes in oesophageal mucosa. *Arch. Pathol.*, **98**, 87.

WORLD HEALTH ORGANIZATION (1977). *Histological Typing of Gastric and Oesophageal Tumours*. International histological classification of tumours, no. 18.

WRIGHT, E.A. (1984). Cell proliferation in health and disease. *Recent Adv. Histopathol.*, **12**, 17.